# Synthesis and Optoelectronic Properties of Threaded BODIPYs

**DOI:** 10.1002/open.202400196

**Published:** 2024-07-23

**Authors:** Matthieu Hicguet, Olivier Mongin, Yann R. Leroux, Thierry Roisnel, Fabienne Berrée, Yann Trolez

**Affiliations:** ^1^ ISCR – UMR6226 École Nationale Supérieure de Chimie de Rennes CNRS ISCR – UMR6226 Univ Rennes F-35000 Rennes France

**Keywords:** boron, fluorescence, BODIPY

## Abstract

We report on the synthesis of two new threaded BODIPYs **5** and **6** in good yields using boron as a gathering atom and a macrocycle with a 2,2’‐biphenol unit. In addition to usual techniques, they were characterized by X‐ray crystallography. Their electrochemical and optical properties were investigated. In particular, both compounds are highly emissive with photoluminescence quantum yields of 54 and 81 % respectively. In addition, they both show a high photostability.

## Graphical abstract

The synthesis of two new threaded BODIPYs in good yields using boron as a gathering atom is reported. In addition to usual techniques, they were characterized by X‐ray crystallography. Their electrochemical and optical properties were investigated. Both compounds are highly emissive with photoluminescence quantum yields up to 81 %.

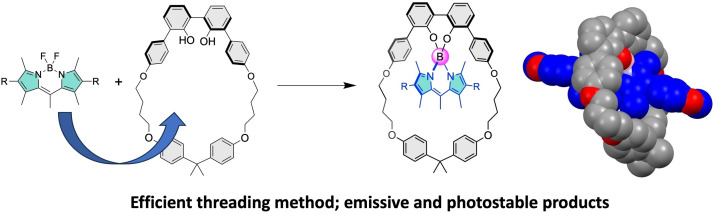

## Introduction

Apart from their usual reactivity in organic synthesis,^[1]^ boron compounds may be used to form complex assemblies such as macrocycles,^[2]^ helicates,^[3]^ cages^[4]^ and (pseudo)‐rotaxanes^[5]^ for instance, furnishing thus functional materials. Among organoboron compounds, tetracoordinate boryl groups are electron‐rich groups and stabilize intermediates with carbon‐boron or heteroatom‐boron bonds.^[6]^ For instance, one archetypal functional compound incorporating tetracoordinated boron is boron dipyrromethene (BODIPY). While its first synthesis was reported in 1968,^[7]^ it is only quite recently that its chemistry found a huge development.^[8]^ The reasons behind this rise are the exceptional fluorescence ability of this family of compounds in addition to high absorption coefficients. These combined optical properties confer them a huge brightness that is particularly suitable for biomedical^[9]^ or more physical applications like organic light‐emitting diodes (OLEDs)^[10]^ or dyes‐sensitized solar cells^[11]^ for instance. Furthermore, these particular features may be tuned with proper functionalization.^[12]^ However, we noticed that the BODIPY unit has scarcely been described as a functional group that can induce the construction of molecular assembly. One impressive example is the pseudorotaxanes synthesized by the group of Nabeshima with a macrocycle incorporating three BODIPYs.^[13]^ Nevertheless, boron does not directly participate to the assembly in this case since the polarization of the B−F bonds is responsible for the threading thanks to their interaction with cationic threads.

**Figure 1:**
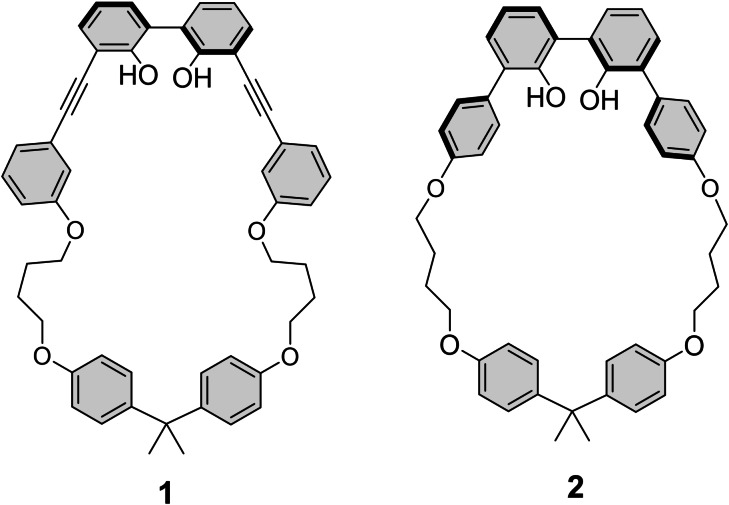
Structures of macrocycles **1** and **2**.

While the boron of BODIPYs is generally substituted with two fluorines, it can also be advantageously substituted by other functional groups to tune their optical properties.^[14]^ Particularly noteworthy is the use of O‐chelates.^[15]^ Actually, we recently described the threading of BODIPYs through a macrocycle bearing a 2,2’‐biphenol unit (compound **1**, Figure 1).^[16]^ The threaded BODIPYs thus exhibited exceptional quantum yields up to 91 % that are significantly higher than their homologs with a simple 2,2’‐biphenol. With the help of theoretical calculations, we showed this difference came from the geometrical constraints. This means that the emissive abilities of the BODIPYs should be very sensitive to the structure of the ligand and in our case the macrocycle. This is the reason why we wanted to investigate another macrocycle, lacking the CC triple bonds (compound **2**, Figure 1), to gain better insights into the structure‐property relationship. In this article, we report on the synthesis of new threaded BODIPYs in good yields. This study reveals significantly different quantum yields for the two species, contrary to what was reported in the first generation of threaded BODIPYs. Although the photoluminescence quantum yields remain high in both cases, this observation shows that a slight change in the structure of the thread could dramatically change the photophysical properties of this kind of species.

## Results and Discussion

**Scheme 1:**
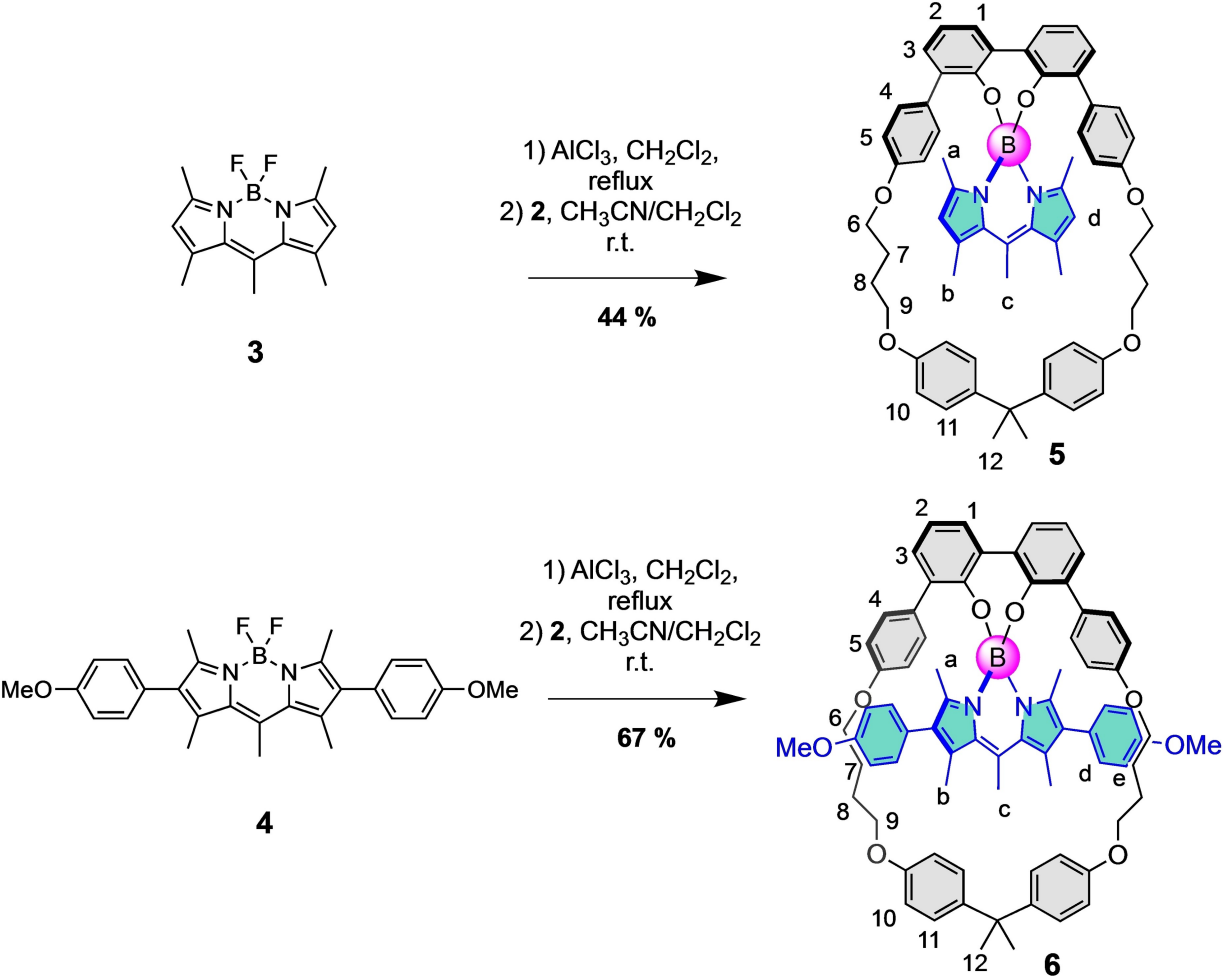
Threading of BODIPYs **3** and **4** through macrocycle **2**.

The synthesis of macrocycle **2** was reported in our recent work.^[16]^ This compound is constituted of a 2,2’‐biphenol unit that is able to coordinate the boron of the BODIPY. The major difference between this macrocycle **2** and macrocycle **1** previously used for the threading of BODIPYs is the absence of triple bonds connected to the biphenol unit. The geometry of macrocycle **2** was supposed to bring more constraint as its internal cavity, whose periphery is constituted of 35 atoms against the 37 atoms of macrocycle **1**, is smaller. BODIPYs **3** and **4** ^[16]^ were threaded through macrocycle **2** following our standard procedure using AlCl_3_ to allow for the substitution of the two fluorines by the 2,2’‐biphenol unit. To do so, BODIPYs **3** and **4** were reacted with AlCl_3_ in refluxing dichloromethane for 2 hours followed by the addition of a solution of macrocycle **2** in acetonitrile and dichloromethane (1/1) at room temperature. After column chromatography, the two threaded species **5** and **6** were isolated in 44 and 67 % yields respectively (Scheme 1). These results are in line with the yields previously reported for similar molecules (31 % to 53 %) with macrocycle **1**.^[16]^ The small improvement observed might be attributed to a greater chemical stability of macrocycle **2** over macrocycle **1** thanks to the absence of the CC triple bond. Actually, we sometimes observed the formation of trace amounts of benzofurans with macrocycle **1** coming from the reaction of the CC triple bond with the adjacent phenol.

**Figure 2:**
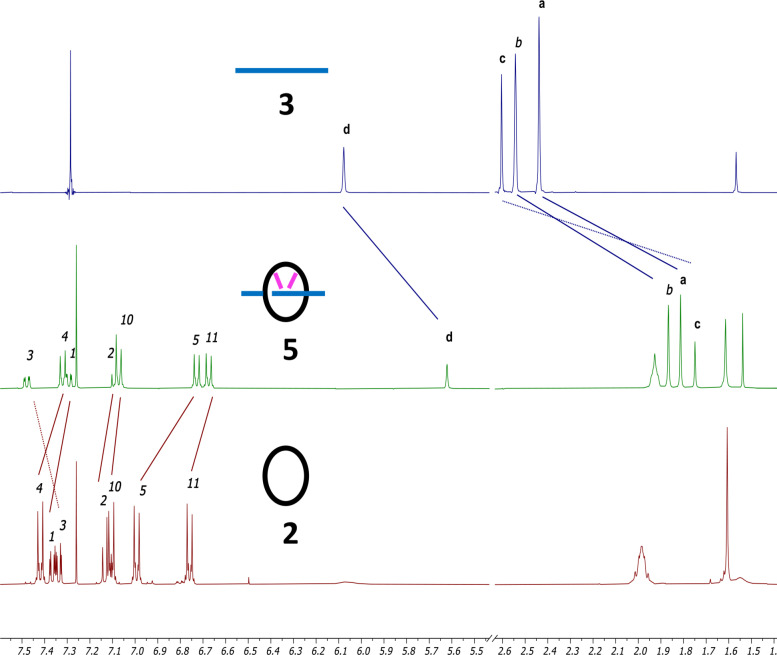
Partial ^1^H NMR spectra (400 MHz, CDCl_3_) of a) BODIPY **3**, b) compound **5** and c) macrocycle **2**. The letters and numbers corresponding to the labelling of the protons are shown in Scheme 1.

Compounds **5** and **6** were characterized by high‐resolution mass spectrometry and NMR spectroscopy. For both compounds, a very strong upfield shift of all protons belonging to the BODIPY cores was observed. For compound **5**, the upfield shifts of the protons a, b and c are comprised between 0.63 and 0.83 ppm and the one of proton d is 0.43 ppm (Figure 2). Such a trend is also observed with compound **6** (Figure S10). The protons of the methyl groups of the BODIPY (a, b and c) are much more impacted (|Δδ|=0.59–0.82 ppm) than the protons of the phenyls d and e (|Δδ|=0.13–0.26 ppm), showing thus the effect of the threading on the chemical shifts. As expected, the chemical shifts of the methoxy groups are almost the same for compounds **4** and **6**, which is logical since these protons are far from the cavity.

**Figure 3:**
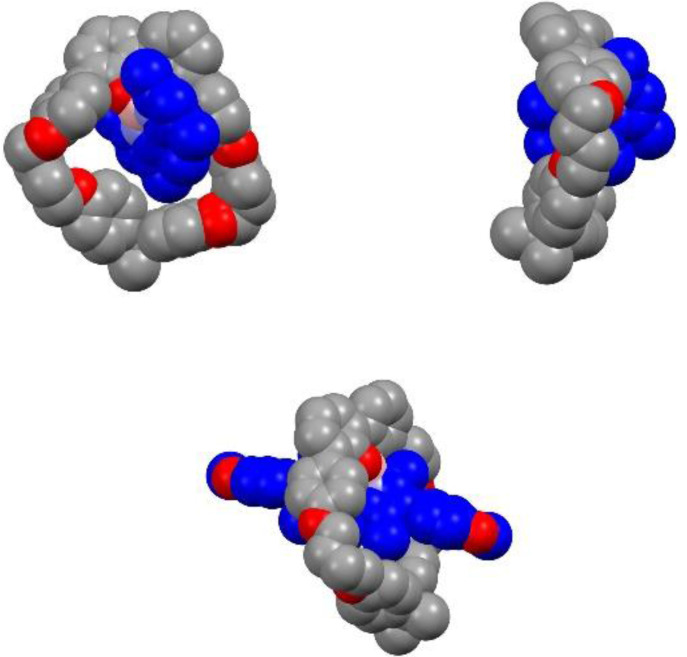
X‐ray structures of compound **5** (top) and compound **6** (bottom) (hydrogens and solvent molecules have been omitted for clarity).

In addition, both compounds **5** and **6** were characterized by X‐ray crystallography. Single crystals suitable for X‐ray diffraction were obtained by slow diffusion of methanol in dichloromethane solutions (Figure 3).^[17]^ These structures definitively confirmed the threaded nature of the BODIPYs that stand inside the cavity, as suggested by ^1^H NMR spectroscopy. Noteworthy is the π‐stacking of the BODIPY with one phenyl of the macrocycle connected to the 2,2’‐biphenol unit. The distance between them is about 3.4 Å in both cases.

**Figure 4:**
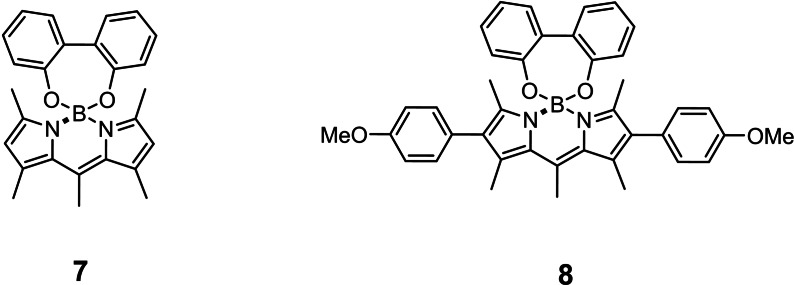
Structure of model compounds **7** and **8**.

**Figure 5:**
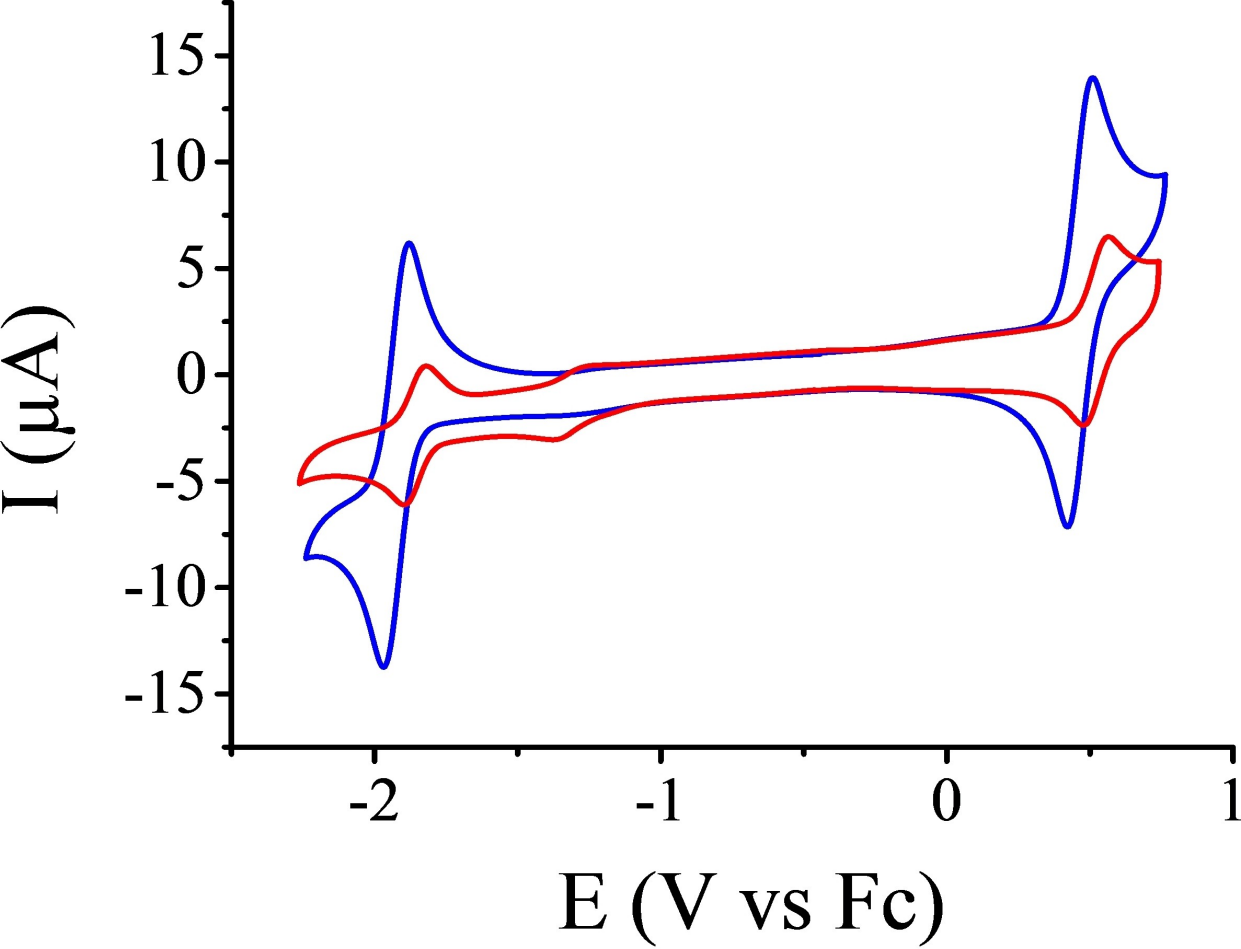
Cyclic voltammograms of compounds **6** (blue curve) and **8** (red curve) in 0.1 M nBu_4_NPF_6_ dichloromethane solution at 0.1 V s^−1^ under Argon. The difference in intensity is due to the different concentrations used to perform the CVs, *i. e*. 1 mM for **6** and 0.35 mM for **8**.

The electrochemical properties of compounds **5** and **6** were evaluated by cyclic voltammetry and compared with their models **7** and **8** ^[16]^ (Figure 4). Both compounds **5** and **7** exhibit a reversible or quasi‐reversible reduction wave at −1.97 and −1.84 V vs Fc respectively (Figure S21). On the anodic side, compound **7** shows several irreversible oxidation waves from +0.5 V vs Fc, while compound **5** shows one quasi‐reversible wave around +0.55 V vs Fc (Figure S22). It is known that BODIPYs without substituents on the β‐positions may oligomerize by oxidations.^[18]^ We hypothesize that this is what happens to compound **7** to explain why the oxidation is irreversible. In the case of compound **5**, the presence of the macrocycle might hamper this oligomerization. This hypothesis is confirmed when considering compounds **6** and **8**. Actually, they exhibit much more reversible waves, especially on the anodic side. While their reduction is very similar to what was observed with compound **5** and **7** with reversible reductions at −1.92 and −1.86 V vs Fc respectively, their oxidations appear at +0.47 and +0.52 V vs Fc (Figure 5). The fact that the oxidation is reversible for both compounds **6** and **8** most probably comes from the presence of the *para*‐methoxyphenyl substituents at the β‐positions of the BODIPYs that prevents them from oligomerizing.

**Figure 6:**
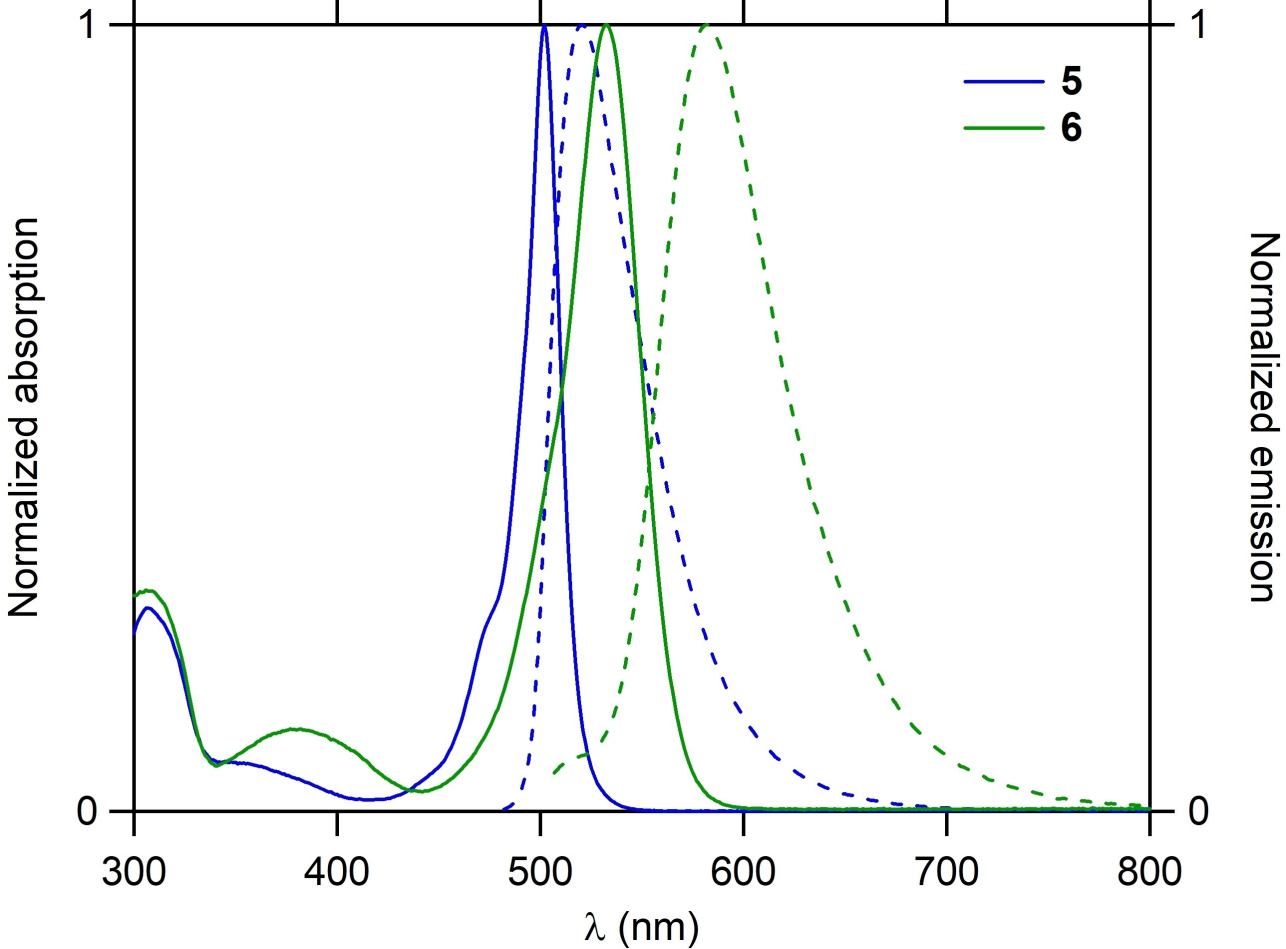
Normalized absorption (straight line) and emission (dashed line) spectra of compounds **5** (blue) and **6** (green) in dichloromethane.

The optical properties of compounds **5** and **6** were also investigated in dichloromethane (Figure 6). They both show an absorption spectrum typical of BODIPYs with a narrow and intense absorption band in the visible region (λ_max_=502 nm and 533 nm respectively). Their emission presents a maximum at 521 and 583 nm respectively with a smaller Stokes shift (726 cm^−1^ and 1609 cm^−1^ respectively) than their models (1028 cm^−1^ and 2055 cm^−1^ respectively) that may be assigned to the high geometrical constraints of the systems (Table 1). Surprisingly, they display significantly different photoluminescence quantum yields (PLQYs). While the PLQY of compound **5** is 54 % (τ=3.4 ns), *i. e*. slightly lower than its model **7** (63 %, τ=4.6 ns), the PLQY of compound **6** is 81 % (6.0 ns), *i. e*. clearly higher than its model **8** (62 %, τ=5.0 ns). The origin of this difference may be explained considering the radiative and nonradiative rate constants (Table 1). Actually, while the radiative rate constants are very close between **5** and **6** (1.6×10^8^ and 1.4×10^8^ s^−1^ respectively), there is a difference of almost one order of magnitude between their nonradiative rate constants (1.4×10^8^ and 3.2×10^7^ s^−1^ respectively). This means that the flexibility of compound **5** is much more important than the one of compound **6**. It probably comes from the presence of the *para*‐methoxyphenyl substituents in compound **6** that limits the movements of the BODIPY inside the macrocycle and thus nonradiative relaxation.

**Table 1:** Photophysical features of compounds **5**–**8**.

Compound	λ_abs_ (nm)	λ_em_ (nm)	Φ_F_	τ (ns)	k_r_ (s^−1^)	k_nr_ (s^−1^)	Stokes shift (cm^−1^)
**5**	502	521	0.54	3.4	1.6×10^8^	1.4×10^8^	726
**6**	533	583	0.81	6.0	1.4×10^8^	3.2×10^7^	1609
**7**	499	526	0.63	4.6	1.4×10^8^	8.0×10^7^	1028
**8**	527	591	0.62	5.0	1.2×10^8^	7.6×10^7^	2055

**Figure 7:**
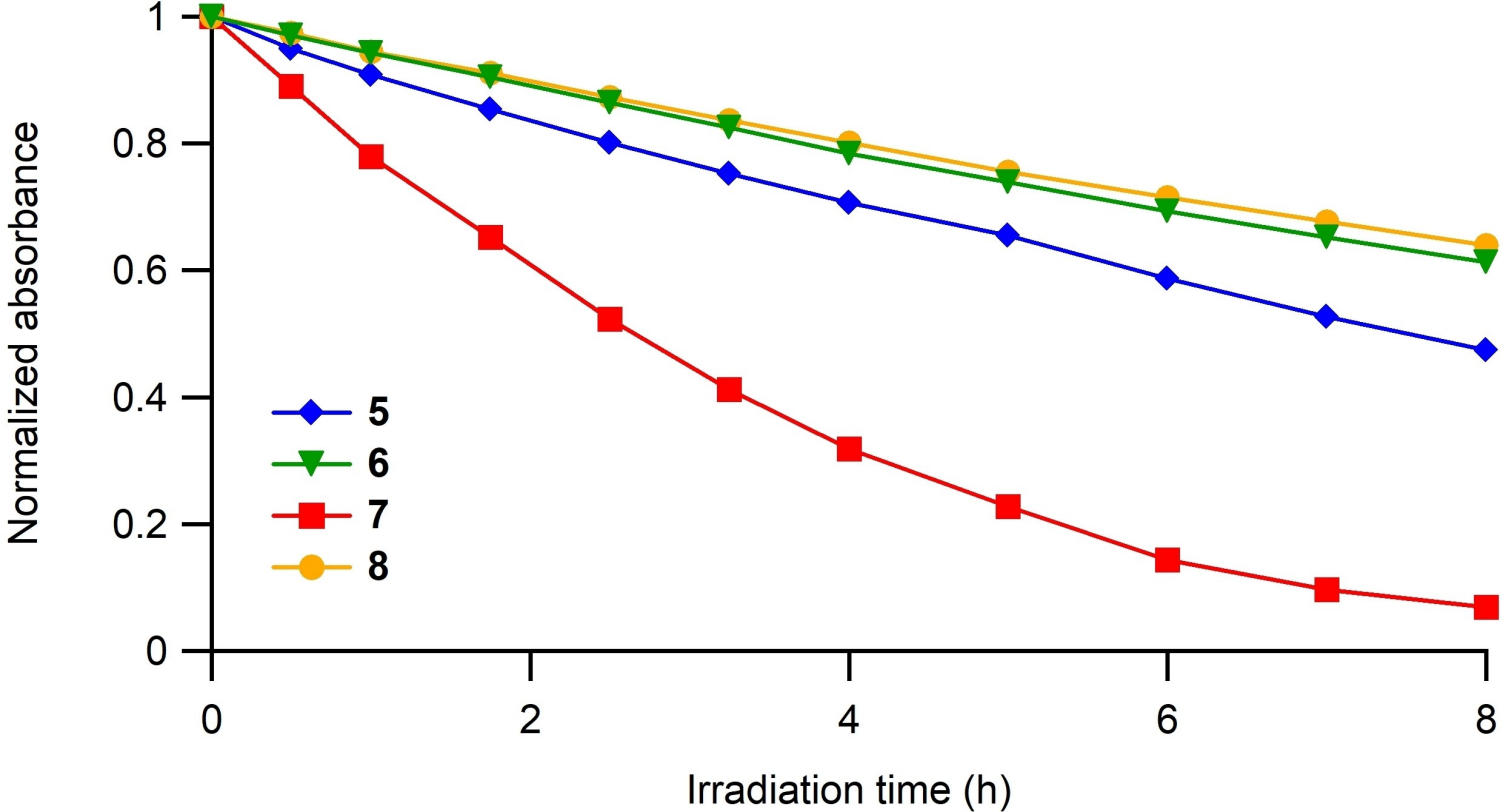
Evolution of the absorbance (normalized at t=0) upon irradiation of compounds **5**–**8** in toluene at room temperature.

The photostability of compounds **5** and **6** was also evaluated and compared with their models **7** and **8**. To do so, we used a lamp sufficiently powerful to induce the degradation of these compounds over hours, followed by UV‐visible spectroscopy (Figures S19 and S20). While compound **5** degraded slower than its model **7**, we were surprised that compound **6** was slightly less photostable than its model **8** (Figure 7). This seems to contradict the beneficial presence of the macrocycle we showed in our previous article. However, to moderate this observation, compounds **6** and **8** are both highly stable, probably thanks to the presence of the *para*‐methoxyphenyl groups compared to compounds **5** and **7**.

## Conclusions

To conclude, we reported the synthesis of two new threaded BODIPYs **5** and **6** in good yields with macrocycle **2**. In addition to usual techniques, they were characterized by X‐ray crystallography. Their electrochemical and optical properties were investigated. In particular, both compounds are highly emissive with PLQYs of 54 and 81 % respectively. In addition, they both show a high photostability. All these features are promising for the future developments of original emissive new materials. Some efforts are currently ongoing in our laboratory to use this methodology to synthesize more complex functional systems.

## Supporting Information

The authors have cited additional references within the Supporting Information.[^19^, ^20^]

## Conflict of Interests

The authors declare no conflict of interest.

1

## Supporting information

As a service to our authors and readers, this journal provides supporting information supplied by the authors. Such materials are peer reviewed and may be re‐organized for online delivery, but are not copy‐edited or typeset. Technical support issues arising from supporting information (other than missing files) should be addressed to the authors.

Supporting Information

## Data Availability

The data that support the findings of this study are available in the supplementary material of this article.
