# Supplementary material for: Synthesis and Optoelectronic Properties of Threaded BODIPYs
Source: ChemistryOpen. 2024 Jul 23;13(11):e202400196. doi: 10.1002/open.202400196 (PMC12056918; doi:10.1002/open.202400196)
Supplement: Supplementary file 1 — Supporting Information [file OPEN-13-e202400196-s001.pdf]

ChemistryOpen

Supporting Information

Synthesis and Optoelectronic Properties of Threaded BODIPYs

Matthieu Hicguet, Olivier Mongin, Yann R. Leroux, Thierry Roisnel, Fabienne Berrée,* and Yann Trolez*

Table of contents

I.	General information	S-2
II.	Synthesis and characterization of the two threaded BODIPYS **5** and **6**	S-3
III.	NMR spectra of new compounds	S-5
IV.	Absorption and emission spectra	S-10
V.	Photostability study	S-14
VI.	Cyclic voltammetry	S-15

I. General Information.

Unless otherwise noted, all solvents and all commercially available chemicals were used without further purification. Anhydrous solvents were purchased from Thermo Scientific and were used without further purification. ^{1}H NMR (400 MHz), ^{13}C NMR (101 MHz) and ^{11}B (128 MHz) spectra were recorded on a Bruker AC 400 spectrometer at 25°C. Chemical shifts δ are given in ppm, and coupling constants *J* in hertz. Multiplicities are presented as follows: s = singlet, d = doublet, t = triplet, m = multiplet, brs = broad singlet. High-resolution mass spectrometry (HRMS) (ESI^+) was performed by the CRMPO, University of Rennes, using a Xevo G2-XS QTof double-focusing mass spectrometer. Purifications by silica gel chromatography were carried out usually on silica 0.040-0.063 mm, 60 Å and by alumina gel chromatography on aluminium oxide, activated, neutral, Brockmann I for compound **5**. Analytical thin-layer chromatography was performed on Merck silica gel 60 F254 plates. Several compounds were purified by flash column chromatography using Geduran® silica gel 60 (0.040-0.063 nm). Melting points were measured on a melting-point apparatus Stuart SMP30 and are uncorrected. Single crystal diffraction data were collected at low temperature on a D8 Venture Bruker AXS CMOS diffractometer with multilayers monochromatized Mo K α radiation. Structures were solved by dual-space algorithm using SHELXT program. All non-hydrogen atoms were refined anisotropically by the full-matrix least-squares techniques using the program SHELXL-2014. Photophysical measurements were performed on freshly-prepared air-equilibrated solutions contained in quartz cells of 1 cm pathlength at room temperature. UV-Vis absorption spectra were recorded on a *J*asco V-770 spectrophotometer. Steady-state fluorescence measurements were performed using a *J*asco FP-8300 fluorimeter equipped with a 150 W Xenon lamp and a Hamamatsu R928 photomultiplier tube. Fully corrected emission spectra were obtained, for each compound, after excitation at the wavelength of the absorption maximum, with $A_{\lambda ex} < 0.1$ to minimize internal absorption. Fluorescein in 0.1 M NaOH (Φ = 0.90 at λ_{ex} = 465 nm) was used as a standard.[19] Fluorescence lifetimes were measured by time correlated single-photon counting (TCSPC) by using an Edinburgh Instrument (FLS920) fluorimeter. Excitation at 466 nm was achieved by a pulsed laser EPL-475. The instrument response (FWHM ca. 1 ns) was determined by measuring the light scattered by a dichloromethane cuvette. The TCSPC traces were analyzed by standard iterative reconvolution methods implemented in the software of the fluorimeter. The compounds displayed strictly monoexponential fluorescence decays (χ^2<1.1). All electrochemical measurements were performed with an Autolab PGSTAT 101 (Metrohm) using a conventional three-electrode cell. A glassy carbon electrode (3mm diameter, IJ Cambria) was used as the working electrode, a platinum wire (1mm diameter, Goodfelloow) as the auxiliary electrode and a KCl saturated calomel electrode (Metrohm) as reference electrode. The glassy carbon electrode was first polished with SiC papers and rinse with water and acetone. Unless otherwise noted, all concentrations of the electrochemically tested molecules are 1 mM.

[19] N. Demas, G. A. Crosby, *J. Phys. Chem.*, **1971**, *75*, 991-1024.

II. Synthesis and characterization of compounds

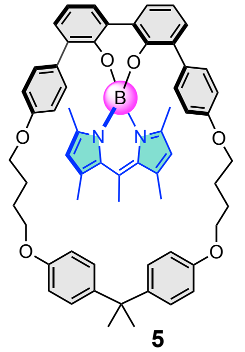

5

To a solution of BODIPY **3** (12 mg, 0.05 mmol) in 2 mL of anhydrous CH_2Cl_2 was added $AlCl_3$ (17 mg, 0.13 mmol) under nitrogen and the resulting mixture was stirred at reflux for 2 hours. The mixture was cooled down to room temperature. **2**[16] (35 mg, 0.05 mmol) in 1 mL of anhydrous CH_3CN and 1 mL of anhydrous CH_2Cl_2 was added and the mixture was stirred overnight at room temperature. The solvent was removed *in vacuo* then purification by alumina chromatography (cyclohexane/CH_2Cl_2: 70/30 to 30/70) afforded **6** (19 mg, 0.02 mmol, 44 %) as an orange solid. Mp: 246°C.

^{1}H NMR (400 MHz, $CDCl_3$) δ 7.50 (dd, *J* = 7.8, 1.8 Hz, 2H), 7.37-7.30 (m, 6H), 7.13-7.05 (m, 6H), 6.80-6.73 (m, 4H), 6.72-6.66 (m, 4H), 5.65 (s, 2H), 4.13-4.05 (m, 4H), 3.90-3.86 (m, 4H), 1.98-1.92 (m, 8H), 1.89 (s, 6H), 1.84 (s, 6H), 1.77 (s, 3H), 1.64 (s, 6H).

^{13}C{^{1}H} **NMR** (100 MHz, $CDCl_3$) δ 156.8, 156.7, 153.5, 152.1, 143.1, 140.3, 139.7, 133.9, 132.8, 132.0, 131.5, 131.0, 129.6, 129.1, 127.6, 121.4, 120.8, 113.6 (2C), 66.8, 66.6, 41.5, 30.9, 25.4, 25.3, 17.3, 16.6, 15.2.

^{11}B{^{1}H} **NMR** (128 MHz, $CDCl_3$) δ 3.0.

HRMS (ESI$^+$): calculated for [$M^{+\cdot}$] $C_{61}H_{61}{}^{11}BN_2O_6$ 928.4617, found 928.4620.

Crystal data (CCDC 2337887): $C_{61}H_{61}BN_2O_6$ M = 928.92 g.mol^{-1}, T = 150(2) K, monoclinic, space groupe = P 2_1/c, a = 12.9267(13) Å, b = 29.559(3) Å, c = 14.7262(16) Å, β = 109.661(4)°, V = 5263.0(10) Å^3, Dc = 1.172 g.cm^{-3}, absorption coefficient = 0.075 mm^{-1}, Final refinement on F^2 with 12081 unique intensities and 645 parameters converged at $\omega R(F^2)$ = 0.1626 (R_F = 0.0604) for 9237 observed reflections with ($I > 2\sigma$).

[16] M. Hicguet, L. Verrieux, O. Mongin, T. Roisnel, F. Berrée, A. Fihey, B. Le Guennic, Y. Trolez, *Angew. Chem. Int. Ed.* **2024**, *63*, e202318297.

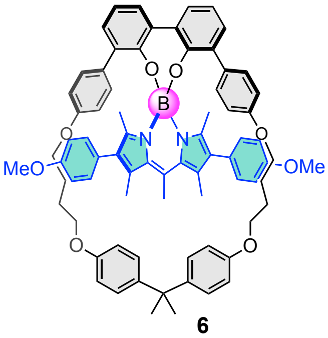

6

To a solution of BODIPY **4**[20] (13 mg, 0.03 mmol) in 2 mL of anhydrous CH_2Cl_2 was added $AlCl_3$ (9 mg, 0.07 mmol) under nitrogen and the resulting mixture was stirred at reflux for 2 hours. The mixture was cooled down to room temperature. **2** (21 mg, 0.03 mmol) in 1 mL of anhydrous CH_3CN and 1 mL of anhydrous CH_2Cl_2 was added and the mixture was stirred overnight at room temperature. The solvent was removed *in vacuo* then purification by alumina chromatography (pentane/CH_2Cl_2: 50/50) afforded **6** (21 mg, 0.02 mmol, 67 %) as a red solid. Mp: 235°C.

^{1}H NMR (400 MHz, $CDCl_3$) δ 7.50 (dd, J = 7.8, 1.7 Hz, 2H), 7.45-7.38 (m, 4H), 7.35 (dd, J = 7.6, 1.7 Hz, 2H), 7.11 (t, J = 7.8 Hz, 2H), 7.02-6.97 (m, 4H), 6.94-6.85 (m, 8H), 6.80-6.75 (m, 4H), 6.55-6.50 (m, 4H), 4.10-4.03 (m, 4H), 3.85 (s, 6H) 3.80-3.71 (m, 4H), 1.96-1.90 (m, 8H), 1.88 (s, 3H), 1.77 (2s, 12H), 1.58 (s, 6H).

^{13}C{^{1}H} **NMR** (100 MHz, $CDCl_3$) δ 158.5, 157.1, 156.8, 152.5, 152.4, 143.2, 140.4, 135.7, 134.1, 133.0, 132.6, 132.1, 131.9, 131.6, 131.4, 129.8, 129.1, 127.7, 126.8, 121.7, 113.8, 113.7 (2C), 66.8, 66.6, 55.4, 41.6, 31.0, 25.4, 25.3, 17.8, 15.5, 14.3

^{11}B{^{1}H} **NMR** (128 MHz, $CDCl_3$) δ 3.4.

HRMS (ESI$^+$): calculated for [$M^{+\cdot}$] $C_{75}H_{73}{}^{11}BN_2O_8$ 1140.5454, found 1140.5456.

Crystal data (CCDC 2337888): $C_{75}H_{73}BN_2O_8$ CH_2Cl_2 M = 1226.09 g.mol^{-1}, T = 150(2) K, monoclinic, space groupe = P 2_1/n, a = 12.2687(6) Å, b = 31.2478(13) Å, c = 33.4874(11) Å, β = 95.777(2) °, V = 12772.9(9) Å^3, Dc = 1.275 g.cm^{-3}, absorption coefficient = 0.162 mm^{-1}, Final refinement on F^2 with 29295 unique intensities and 1604 parameters converged at $\omega R(F^2)$ = 0.2070 (R_F = 0.0860) for 23306 observed reflections with ($I > 2\sigma$).

[20] W. Ren, H. Xiang, C. Peng, Z. Musha, J. Chen, X. Li, R. Huang, Y. Hu, *RSC Adv.*, **2018**, *8*, 5542-5549.

III. NMR spectra of new compounds

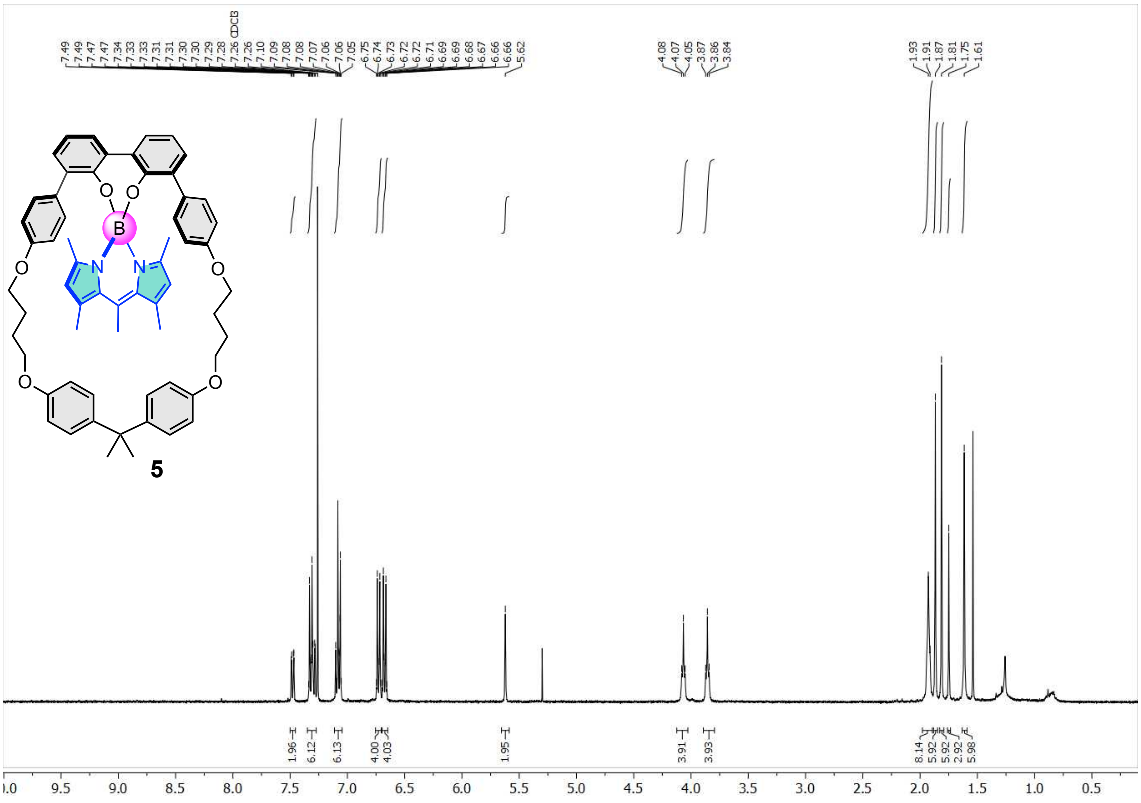

Figure S1. ^{1}H NMR spectrum (400 MHz, $CDCl_3$) of compound **5.**

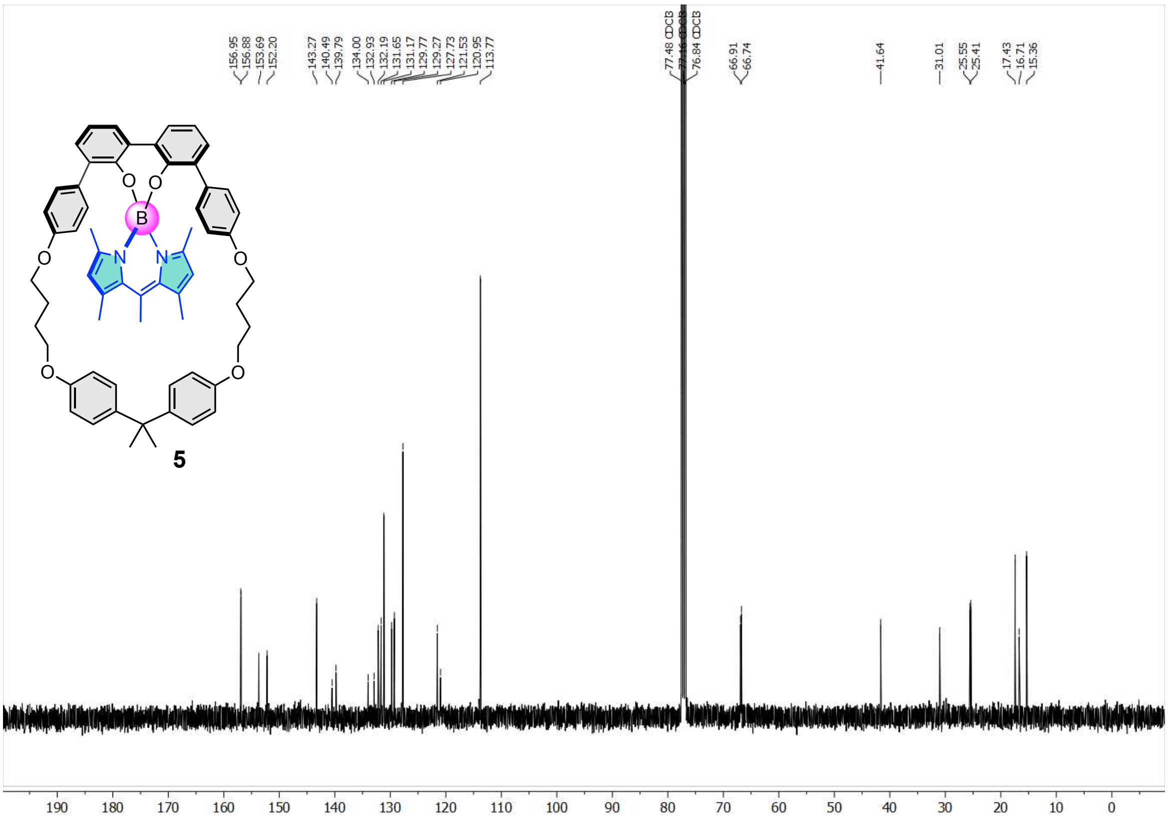

Figure S2. $^{13}C\{^{1}H\}$ NMR spectrum (100 MHz, $CDCl_3$) of compound **5.**

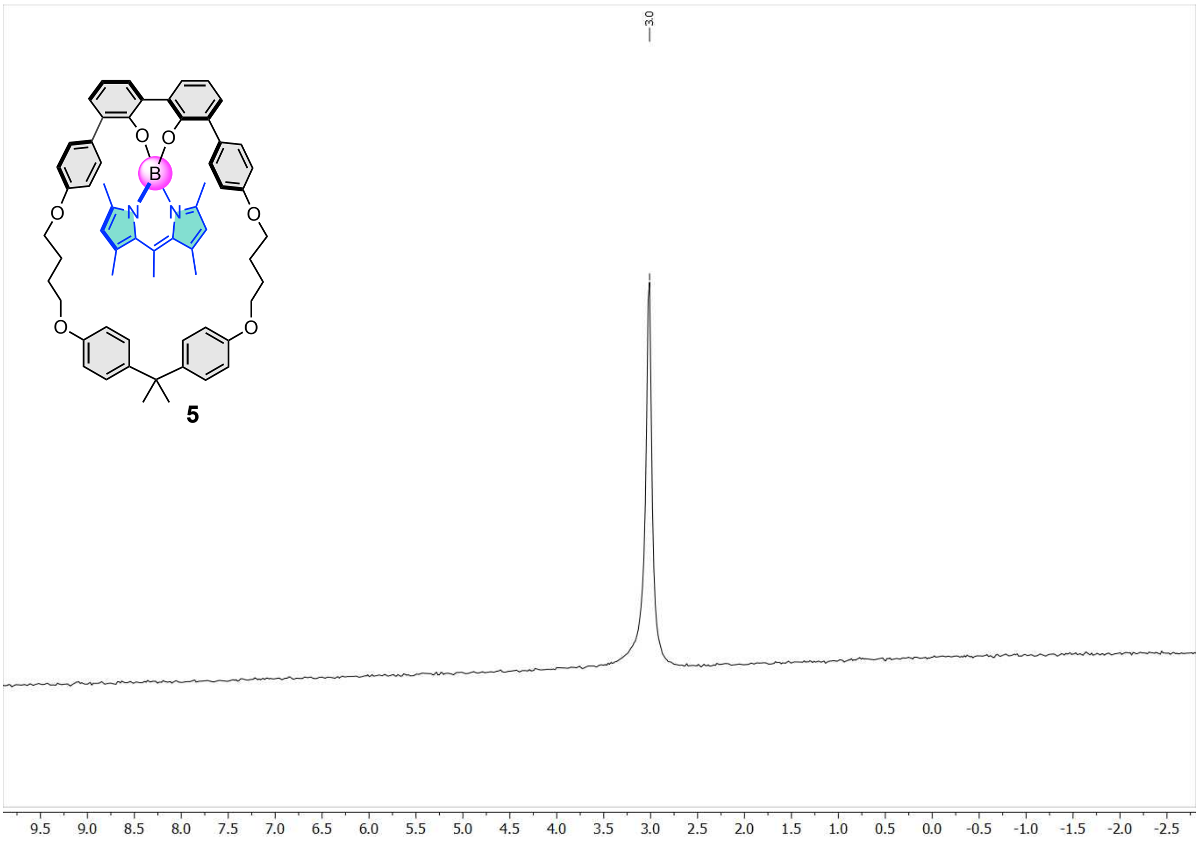

Figure S3. $^{11}B\{^{1}H\}$ NMR spectrum (128 MHz, $CDCl_3$) of compound **5**.

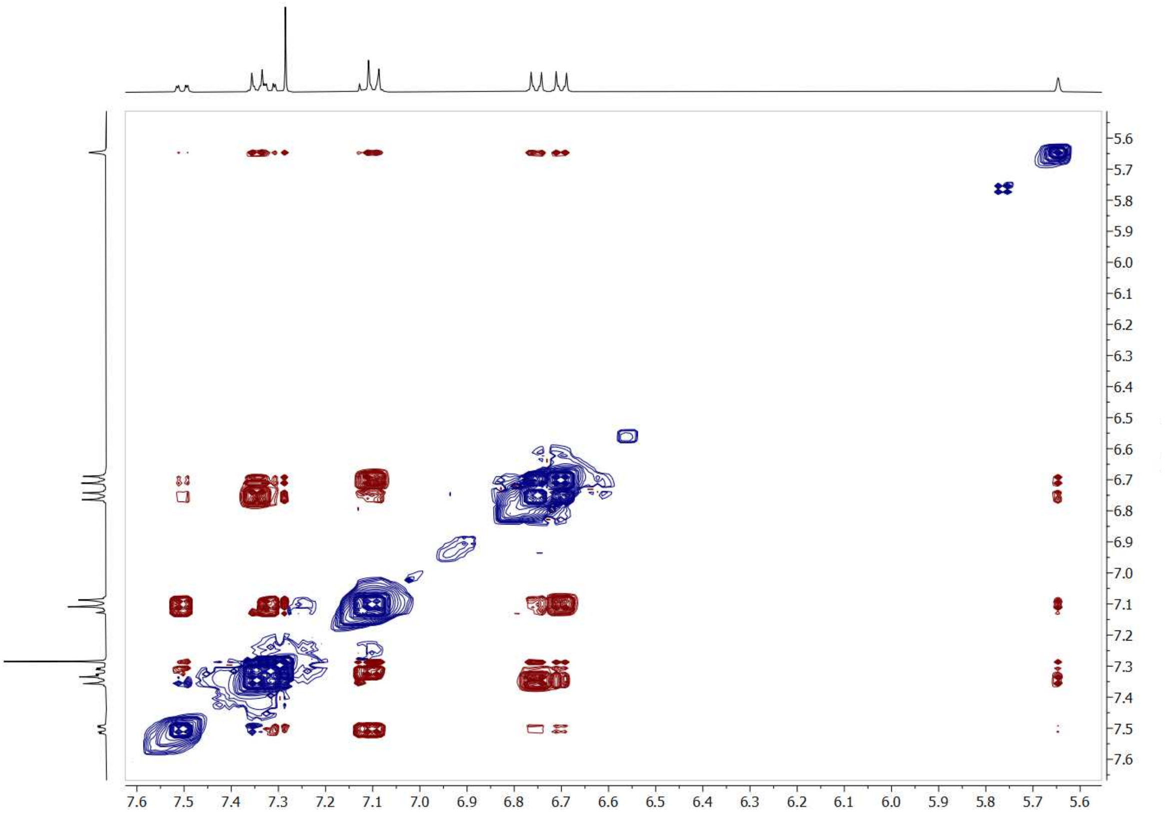

Figure S4. ^{1}H NOESY NMR spectrum (400 MHz, $CDCl_3$) of compound **5** in the range 5.6 to 7.6 ppm.

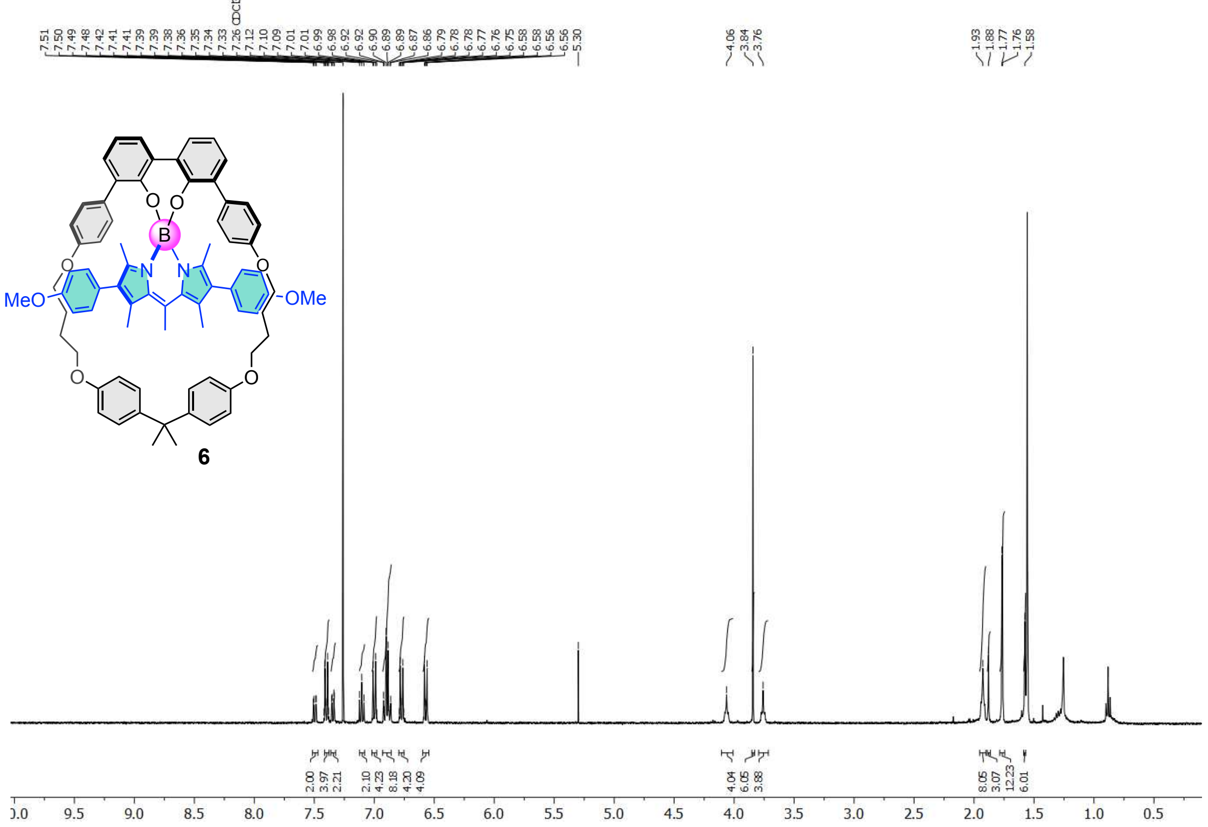

Figure S5. ^{1}H NMR spectrum (400 MHz, $CDCl_3$) of compound **6.**

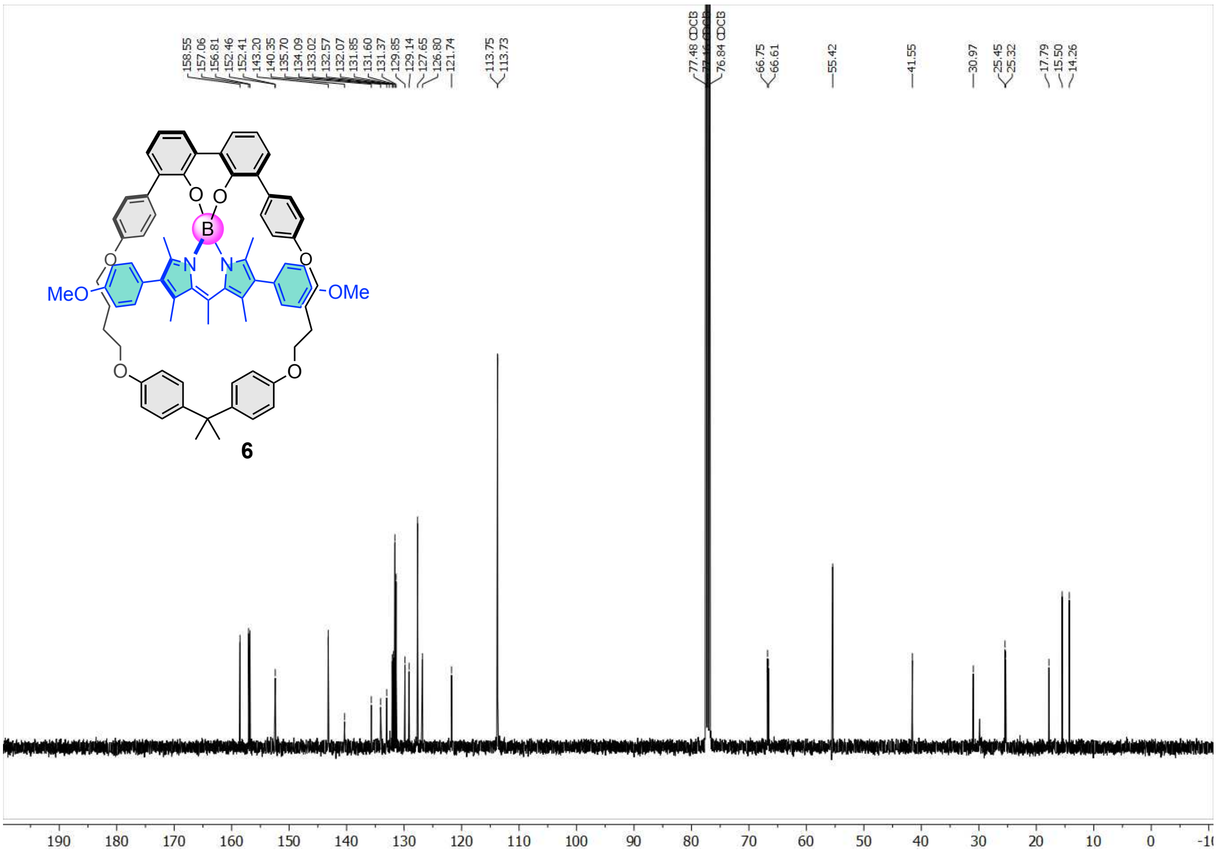

Figure S6. $^{13}C\{^{1}H\}$ NMR spectrum (100 MHz, $CDCl_3$) of compound **6.**

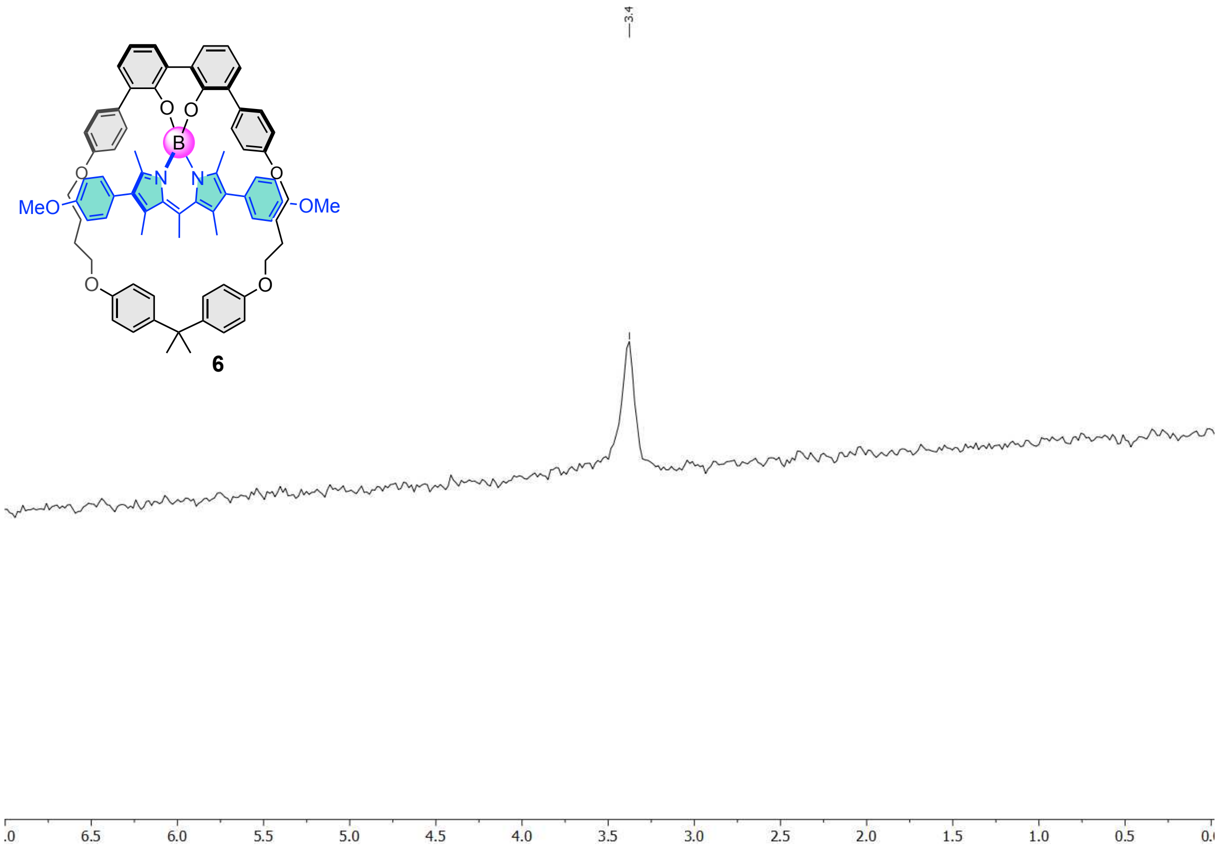

Figure S7. $^{11}B\{^{1}H\}$ NMR spectrum (128 MHz, $CDCl_3$) of compound **6**.

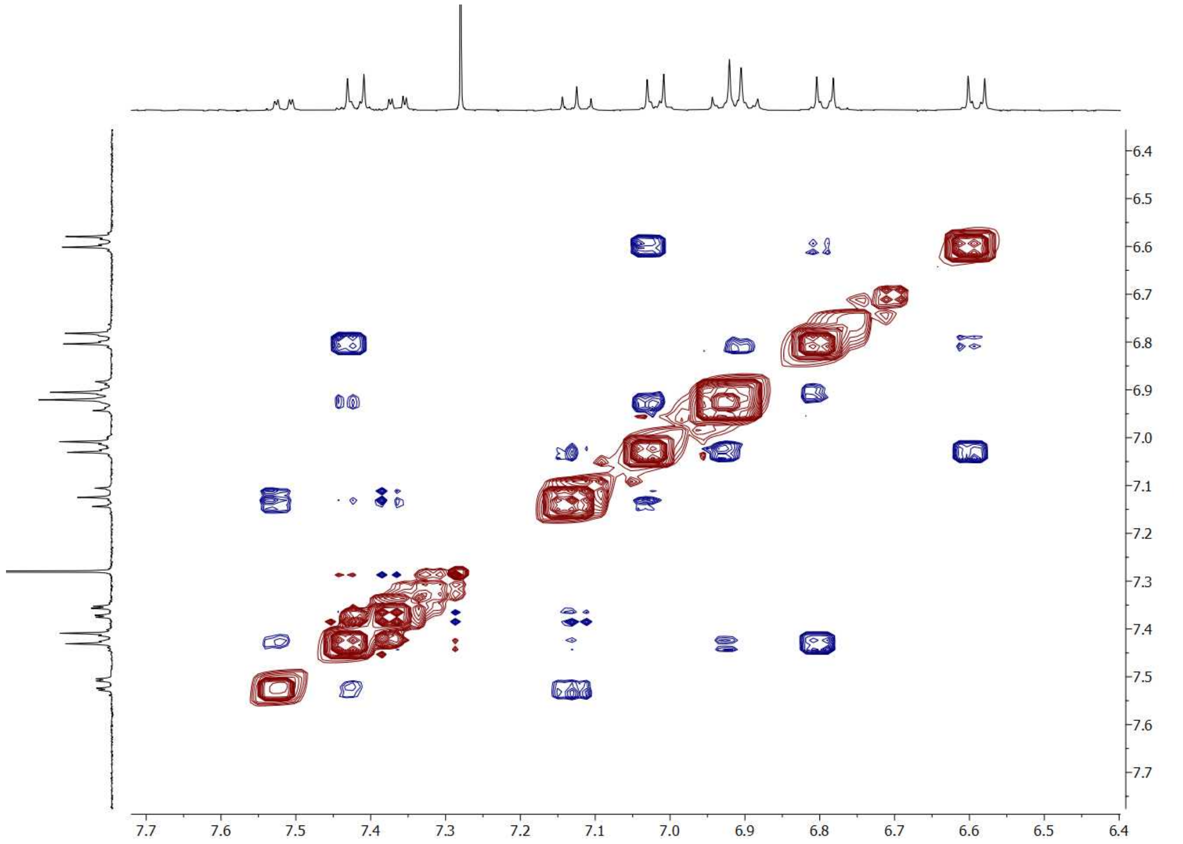

Figure S8. ^{1}H NOESY NMR spectrum (400 MHz, $CDCl_3$) of compound **6** in the range 6.4 to 7.7 ppm.

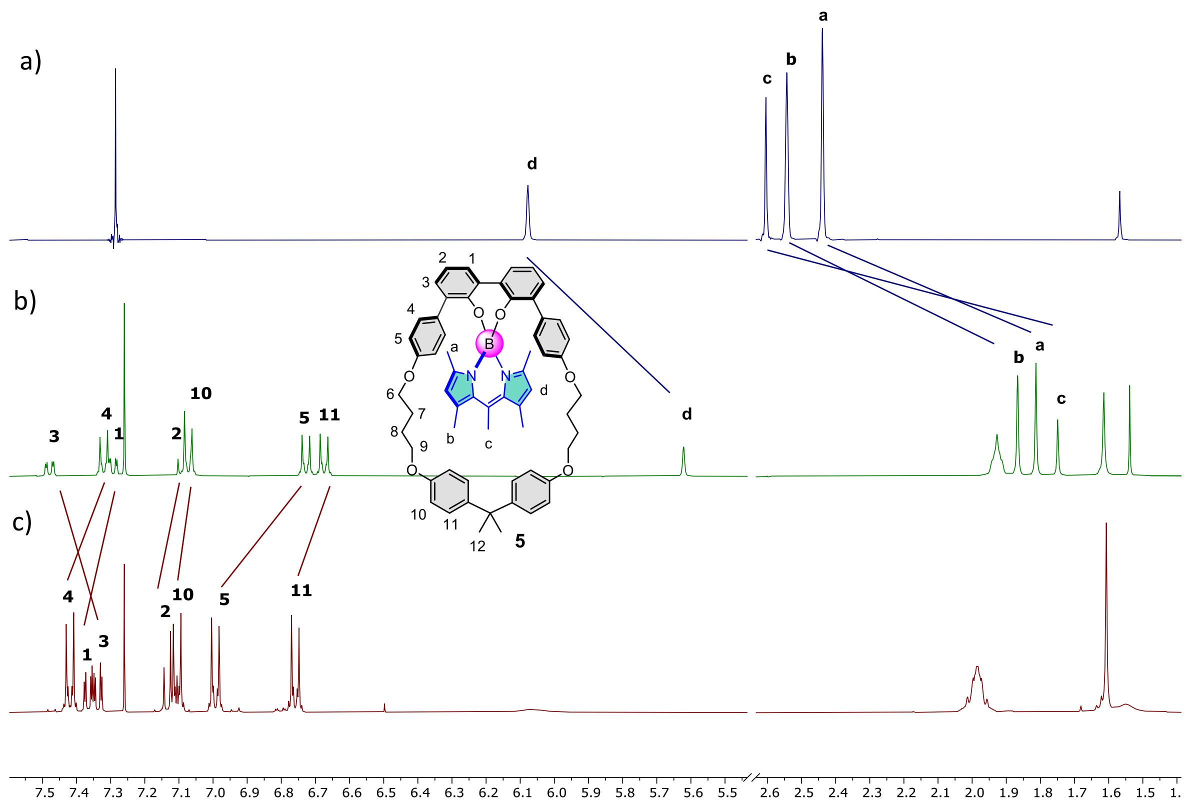

Figure S9. Partial ^{1}H NMR spectra (400 MHz, $CDCl_3$) of a) BODIPY **3**, b) compound **5** and c) macrocycle **2**.

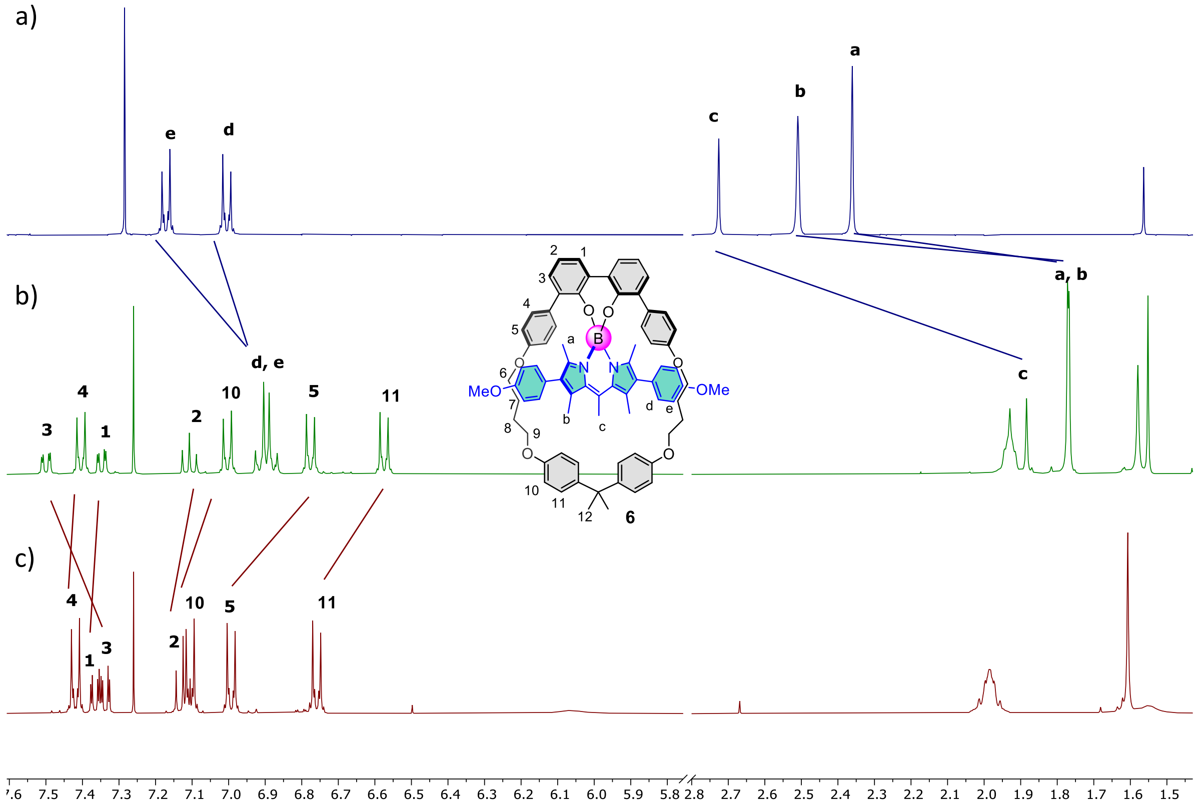

Figure S10. Partial ^{1}H NMR spectra (400 MHz, $CDCl_3$) of a) BODIPY **4**, b) compound **6** and c) macrocycle **2**.

IV. Absorption and emission spectra.

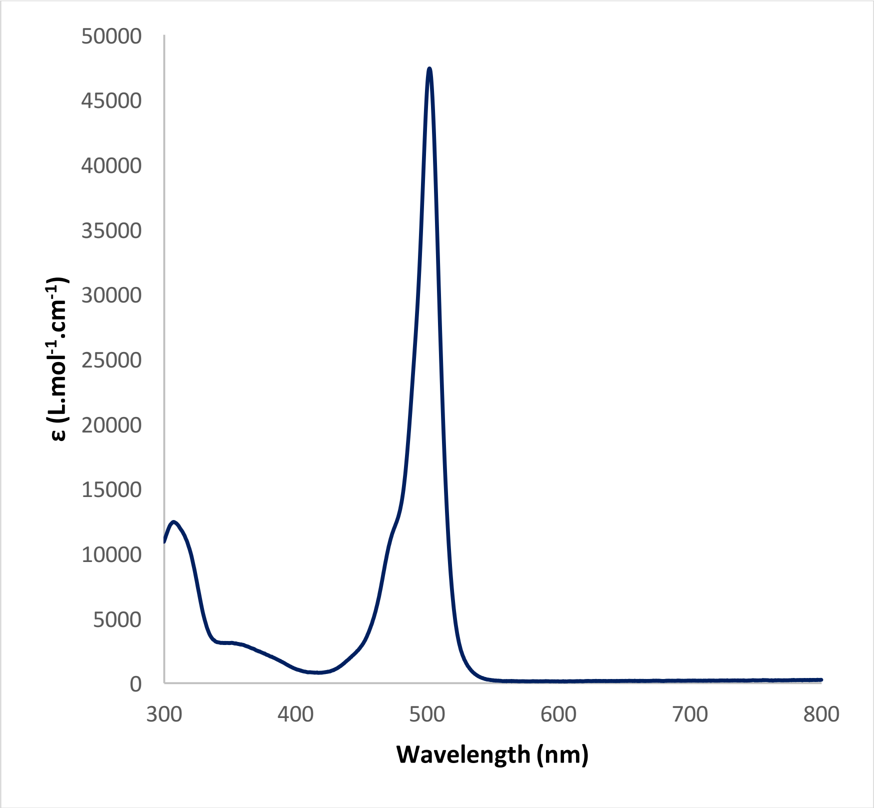

Figure S11. Absorption spectrum of compound **5** in dichloromethane.

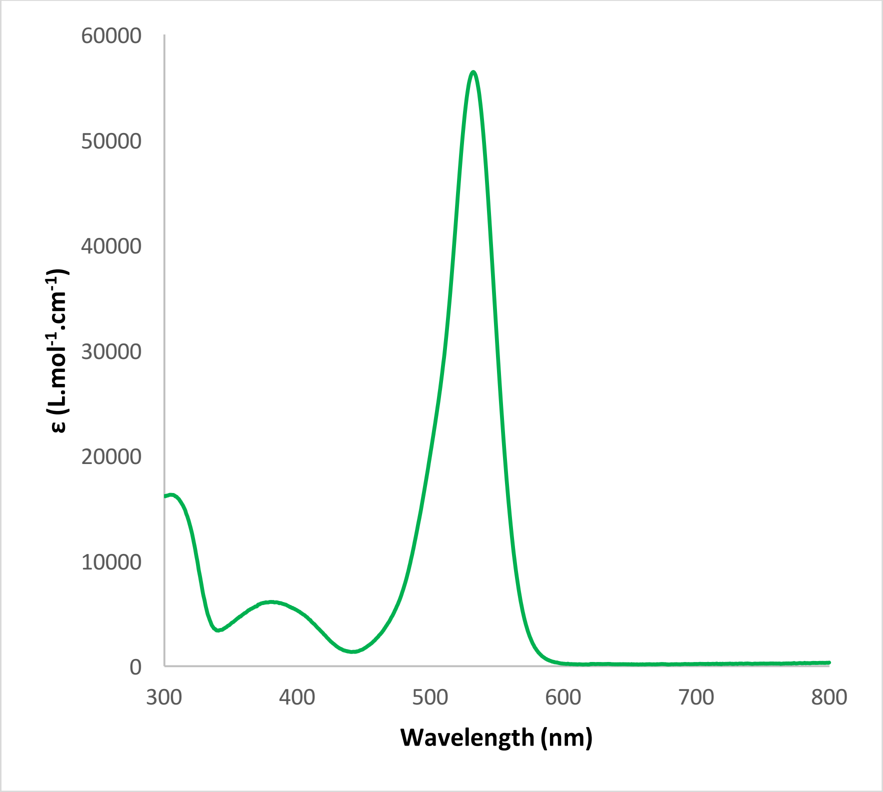

Figure S12. Absorption spectrum of compound **6** in dichloromethane.

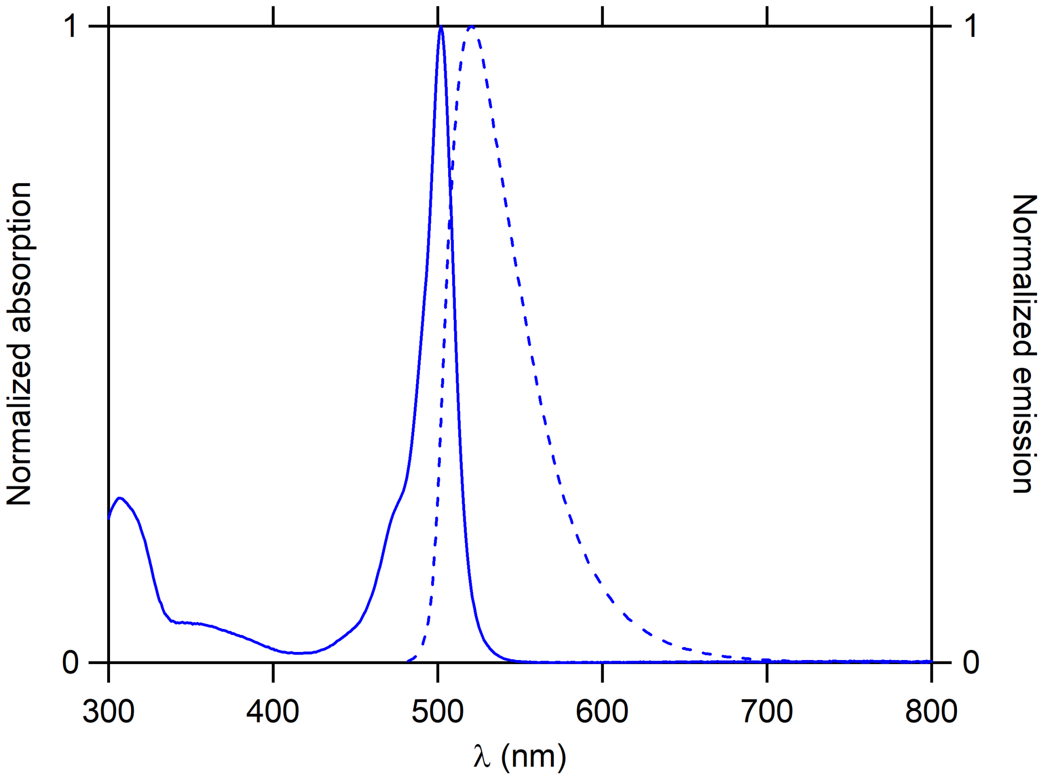

Figure S13. Normalized absorption and emission spectra of compound **5** in dichloromethane.

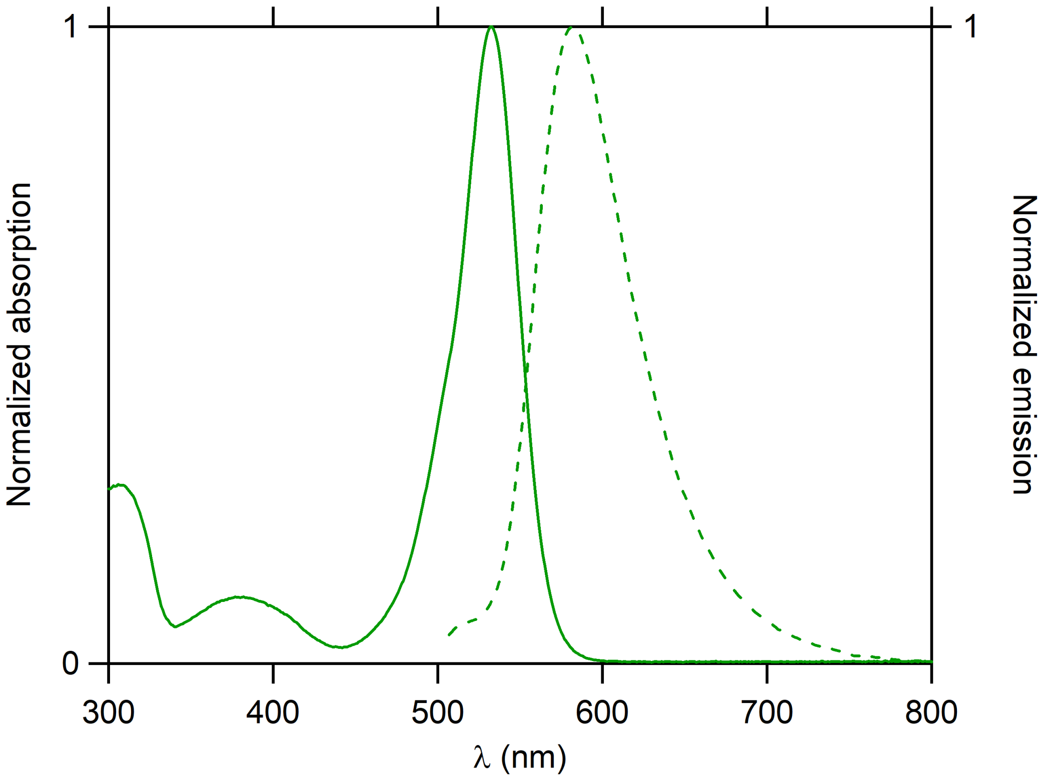

Figure S14. Normalized absorption and emission spectra of compound **6** in dichloromethane.

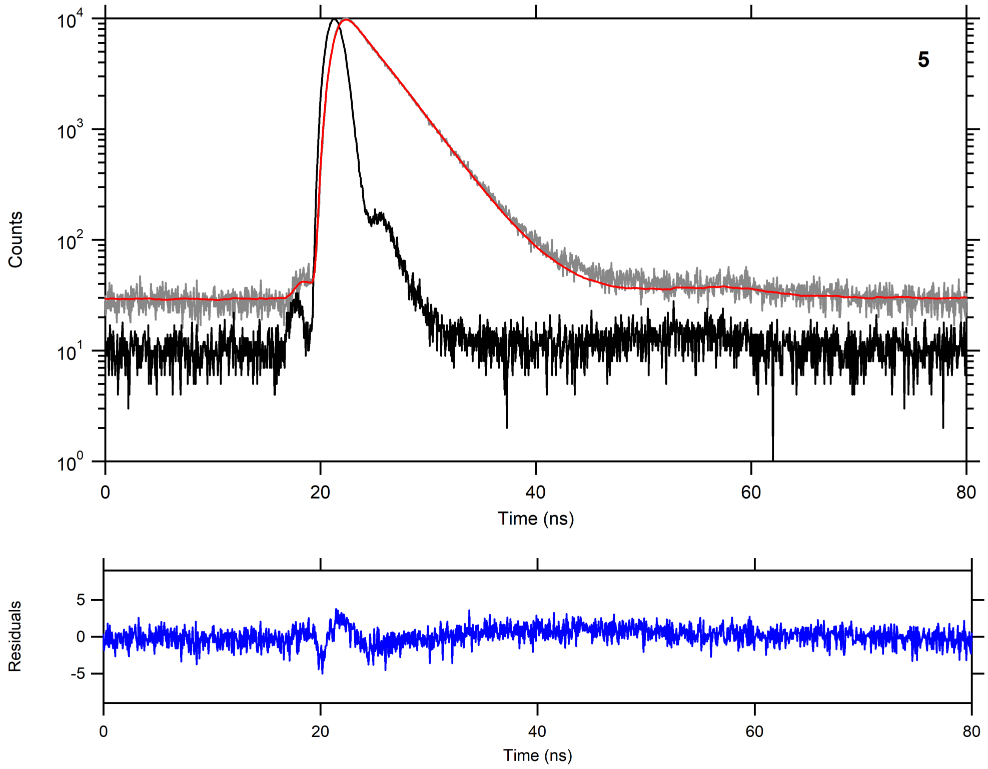

Figure S15. TCSPC data of **5** in dichloromethane (grey curve: measured decay; black curve: IRF; red curve: fitted decay; blue curve: residuals).

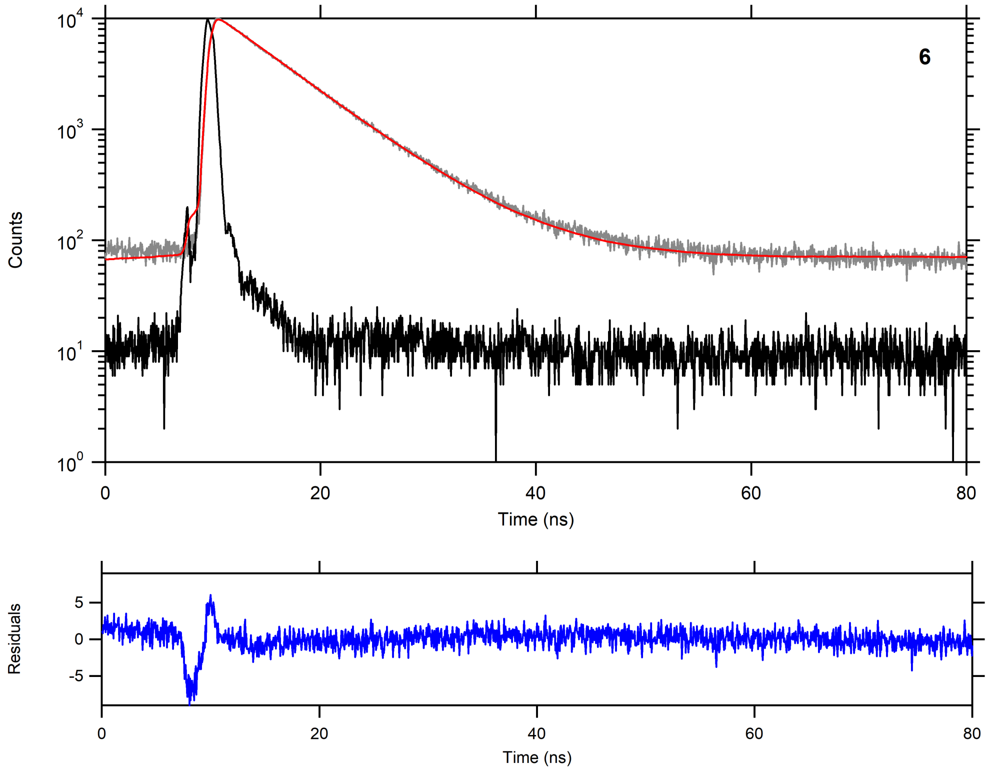

Figure S16. TCSPC data of **6** in dichloromethane (grey curve: measured decay; black curve: IRF; red curve: fitted decay; blue curve: residuals).

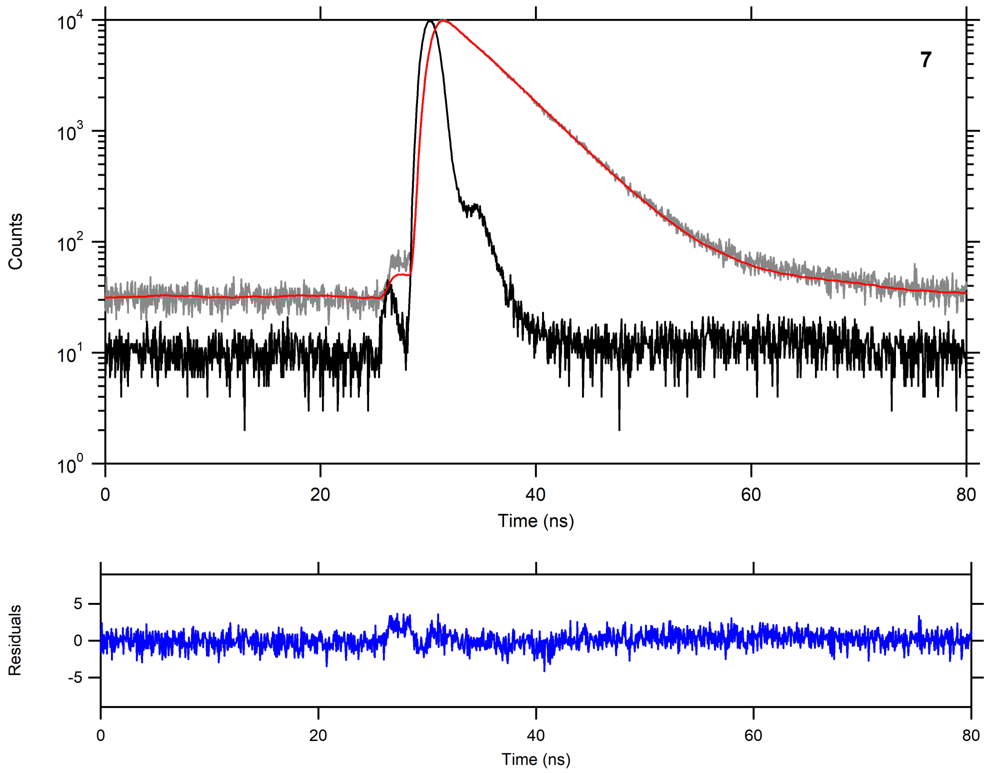

Figure S17. TCSPC data of **7** in dichloromethane (grey curve: measured decay; black curve: IRF; red curve: fitted decay; blue curve: residuals).

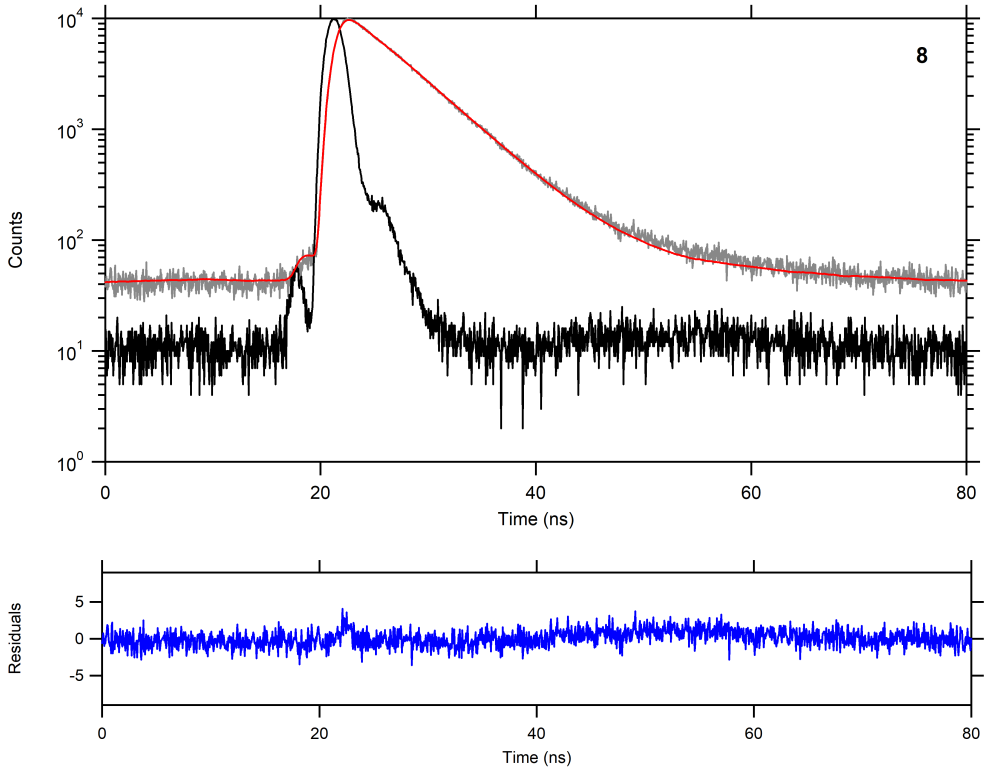

Figure S18. TCSPC data of **8** in dichloromethane (grey curve: measured decay; black curve: IRF; red curve: fitted decay; blue curve: residuals).

V. Photostability study

The photostability study was performed in standard quartz cuvettes (1 cm × 1 cm). The solutions of **5** and **6** in air-equilibrated toluene were irradiated at room temperature with a 100 W (10000 lumens) floodlight (15 cm in width by 10 cm in height), consisting in a plate of 96 SMD2835 LEDs. The distance between the cuvettes and the floodlight was 10 cm. The photodegradation of the solutions was followed over 8 hours by monitoring the evolution of their UV-visible spectra. The temperature at the samples was kept below 298 K during the study.

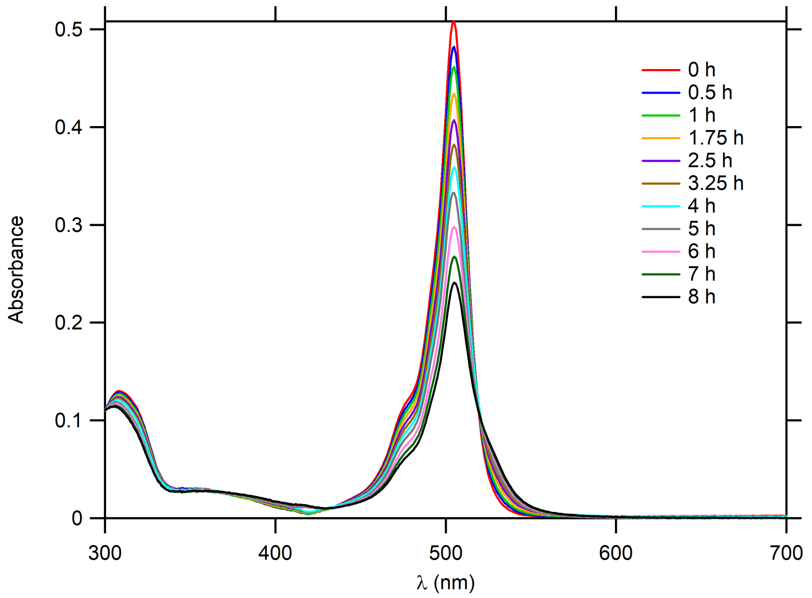

Figure S19. Modification upon irradiation of the absorption spectra of **5** in air-equilibrated toluene at room temperature.

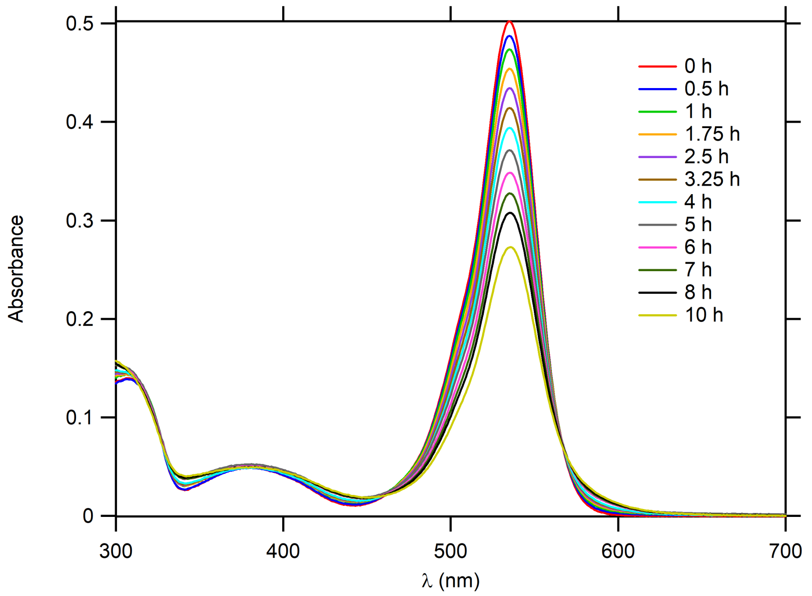

Figure S20. Modification upon irradiation of the absorption spectra of **6** in air-equilibrated toluene at room temperature.

VI. Cyclic voltammetry.

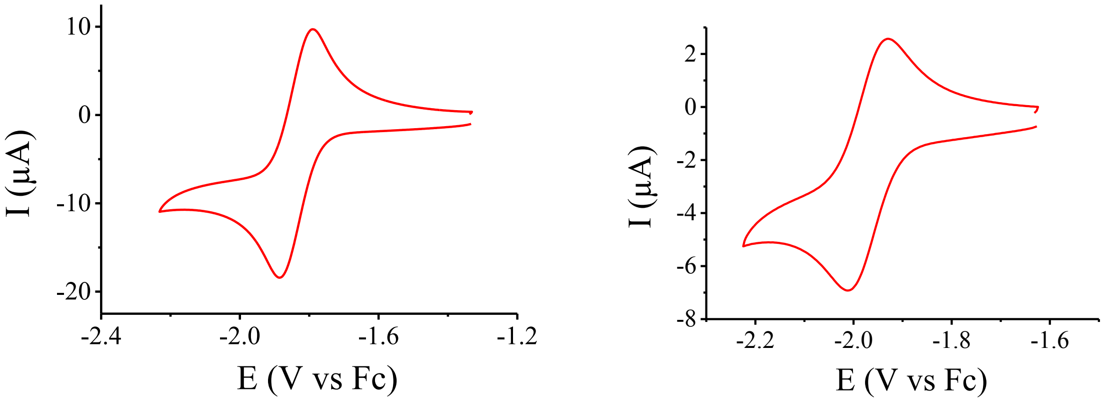

Figure S21. Cyclic voltammograms in reduction of **7** (left) and **5** (right) in a 0.1 m nBu_4NPF_6 dichloromethane solution at 0.1 V s^{-1}.

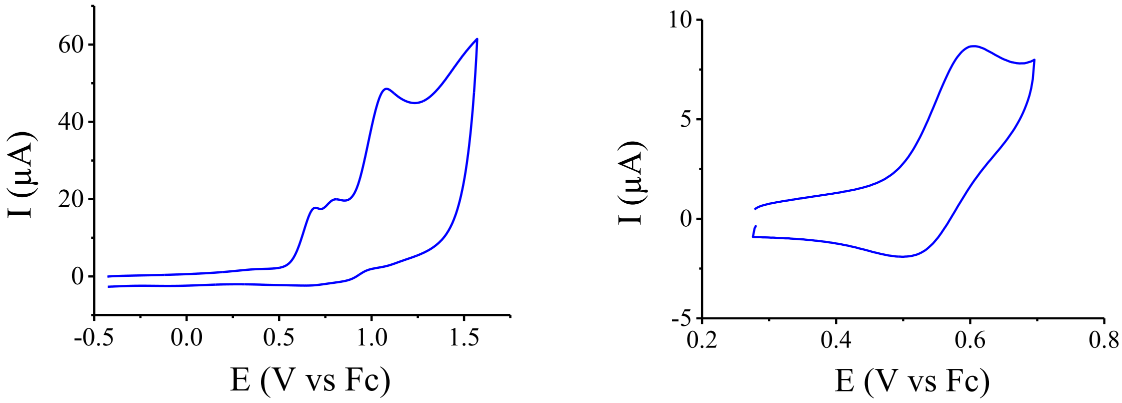

Figure S22. Cyclic voltammograms in oxidation of **7** (left) and **5** (right) in a 0.1 m nBu_4NPF_6 dichloromethane solution at 0.1 V s^{-1}.

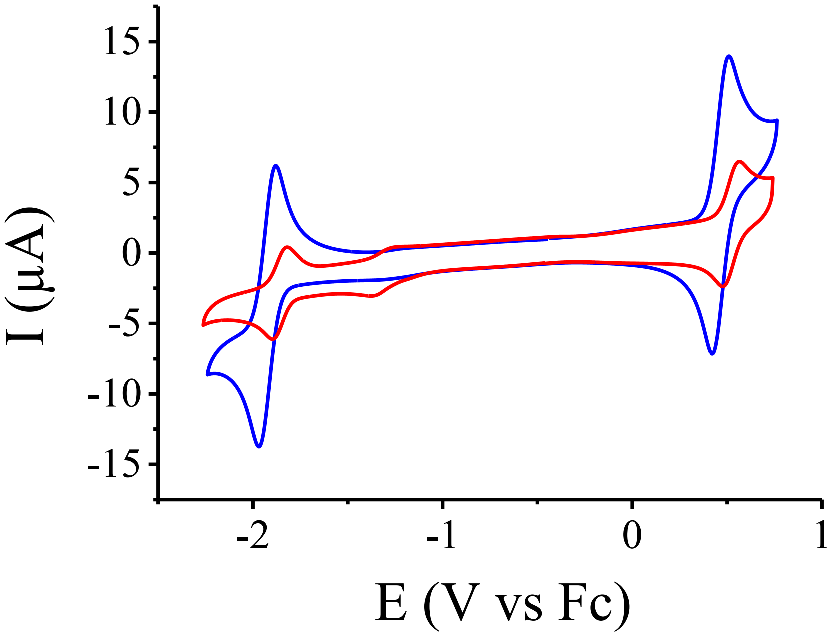

Figure S23. Cyclic voltammograms of **6** (blue curve) and **8** (red curve) in 0.1 M $n\mathrm{Bu_4NPF_6}$ dichloromethane solution at 0.1 V s^{-1} under Argon. The difference in intensity is due to the different concentrations used to perform the CVs, i.e. 1 mM for **6** and 0.35 mM for **8**.
